# Supplementary material for: Feature-temporal predictions dynamically modulate performance, feature-based attentional capture, and motor response activity during visual search
Source: Vis cogn. Author manuscript; Available in PMC 2026 Oct 9. (PMC7619539; doi:10.1080/13506285.2026.2645176)
Supplement: Supplementary file 1 [file EMS219689-supplement-Supplementary_Materials.docx]

**Supplementary Materials**

**Supplementary Figure 1**

Normalised rectified muscle activity in hands used to respond to the likely-early and likely-late targets over the 5500 ms duration trials where both targets appeared, across all learning blocks, recorded using EMG. The solid pink and blue lines represent the mean values and the shaded areas around them represent ±1 SE. The pink and blue columns indicate the time windows over which the likely-early and likely-late targets, respectively, appeared. The two black horizontal lines mark clusters of significant differences in activity between the two hands, where *** = *p* < .001. For the earlier cluster, the peak difference occurred at 1804 ms. For the later cluster, the peak difference occurred at 4717 ms.

**Supplementary Figure 2**

(A) Mean hits for identifying targets in the testing block, for likely-early and likely-late targets appearing at each onset time. Error bars represent ±1 standard error (SE) around the mean, **individual participants are represented as light grey lines.** A 2x2 ANOVA analysing the effects of target likelihood and onset time revealed that neither factor, nor their interaction, significantly affected hits. The full output of this ANOVA is shown in Supplementary Table 1 below. (B) Mean response times (RTs) for identifying targets in the testing block, for likely-early and likely-late targets appearing at each onset time. A 2x2 (target likelihood x onset time) ANOVA revealed a significant main effect of target likelihood on RTs (*p* < .001). Overall, participants were significantly faster to identify the likely-early target (M = 896ms, SD = 49ms) than the likely-late target (M = 949ms, SD = 49ms). The ANOVA also revealed a significant interaction between target likelihood and onset time (*p* < .001). A follow-up t-test revealed that, when targets appeared at 1000 ms, target likelihood significantly affected RTs (*t*(47) = -6.94, *p* < .001, *d* = -1.00, BF_10_ = 1262412.00) whereby the likely-early target (M = 891ms, SD = 69ms) was identified significantly faster than the likely-late target (M = 989ms, SD = 69ms). A second follow-up t-test revealed that, when targets appeared at 4000 ms, target likelihood had no significant effect on RTs (*t*(47) = 0.33, *p* = .746, *d* = 0.05, BF_10_ = 0.16). The statistical significance of comparisons is indicated in the figure with asterisks: *** = *p* < .001, n.s. = not significant. Full output of the ANOVA analysing RTs is shown in Supplementary Table 2 below.

**Supplementary Table 1**

Hits ANOVA Output

| Predictor | *F* | *df_1_* | *df_2_* | *p* | *η*^2^_G_ |
| --- | --- | --- | --- | --- | --- |
| Onset Time | 0.64 | 1 | 47 | .429 | .002 |
| Target Likelihood | 1.49 | 1 | 47 | .228 | .011 |
| Onset Time : Target Likelihood | 1.07 | 1 | 47 | .305 | .004 |

**Supplementary Table 2**

RT ANOVA Output

| Predictor | *F* | *df_1_* | *df_2_* | *p* | *η*^2^_G_ |
| --- | --- | --- | --- | --- | --- |
| Onset Time | 3.46 | 1 | 47 | .069 | .010 |
| **Target Likelihood** | **17.89** | **1** | **47** | **< .001** | **.064** |
| **Onset Time : Target Likelihood** | **26.02** | **1** | **47** | **< .001** | **.078** |

*Note.* Significant predictors are shown in bold.

**Supplementary Table 3**

Colour Type Custom Contrasts for Distractor Fixations Analysis

| Colour Type Level | Goal Dependent Contrast  (No Colour Match vs.  Target Colour Match) | | Prediction Contrast  (Likely-Early vs. Likely-Late Target Colour Match) |
| --- | --- | --- | --- |
| No Match | | 1 | 0 |
| Likely-Early Target Match | | -0.5 | -0.5 |
| Likely-Late Target Match | | -0.5 | 0.5 |

*Note.* The above contrasts were designed to explore two specific comparisons of the three levels of the colour type variable. The levels code whether a distractor shared its colour with neither target (No Match), the likely-early target (Likely-Early Target Match) or the likely-late target (Likely-Late Target Match). The first, ‘goal dependent’, comparison (No Colour Match vs. Target Colour Match) compared distractors which did not share their colour with either target to those which shared their colour with any target. The second, ‘prediction’, comparison (Likely-Early vs. Likely-Late Target Colour Match) compared distractors which shared their colour with the likely-early target to those which shared their colour with the likely-late target.

**Supplementary Figure 3**

(A) Visualisation of the fixed effects output of the GLMM giving the predicted percentage of distractors fixated during target absent trials across levels of colour type, onset times, and all learning blocks. (B) The actual recorded percentage of distractors fixated across levels of colour type, onset times, and all learning blocks. This plot only includes data from target absent trials. The solid pink, blue, and orange lines represent the mean values, while the grey shaded areas represent ±1 SE around the mean. For this plot, data were smoothed by rounding onset times to the nearest 400 ms.
